# Body composition among 6–7 years-old children: data from the 2015 Pelotas (Brazil) Birth Cohort Study

**DOI:** 10.1186/s12887-026-06654-4

**Published:** 2026-03-05

**Authors:** Thaynã Ramos Flores, Otávio Amaral de Andrade Leão, Marlos Rodrigues Domingues, Pedro C. Hallal, Mariângela Freitas da Silveira, Andréa Dâmaso Bertoldi

**Affiliations:** 1 Department of Health and Kinesiology, University of Illinois – Urbana/Champaign, 1206 S 4th St Champaign, Urbana/Champaign, Illinois 61820-6953 USA; 2 Postgraduate Program in Physical Education, Federal University of Pelotas, Pelotas, Brazil; 3 Postgraduate Program in Epidemiology, Federal University of Pelotas, Pelotas, Brazil

**Keywords:** Body composition, Fat mass, Lean mass, Children, Longitudinal studies

## Abstract

**Background:**

Nutritional assessment of children is essential for health, especially given the observed worldwide increase in childhood overweight and obesity. This study aims to describe body composition (fat and lean mass) in children aged 6–7 from the 2015 Pelotas (Brazil) Birth Cohort.

**Methods:**

Longitudinal study that measured fat mass (FM% and FM in kilograms) and lean mass (LM% and LM in kilograms) using Dual-Energy X-ray Absorptiometry (DEXA) and Air-Displacement Plethysmography (BodPod). Body composition was described in relation to the independent variables. Student t-tests were used for binary variables, and ANOVA was used for categorical and ordinal variables.

**Results:**

Of the 4,275 children included in the original cohort, 2,271 had DEXA, and 2,181 had BodPod data at age 6–7 years. Overall, BodPod and DEXA measures presented similar results, with girls having a higher average fat mass percentage in BodPod [25.2% (95%CI 24.7; 25.7) and DEXA [25.9% (95%CI 25.3; 26.4)]. Boys had a higher average lean mass percentage for both equipment [BodPod: 80.5% (95%CI 79.9; 81.1) and DEXA: 74.6% (95%CI 74.1; 75.2)].

**Conclusion:**

Children classified as obese, according to BMI-for-age z-score, had a higher average fat mass percentage (DEXA: 37.2% and BodPod: 34.5%), and this mean was higher among girls. The FM% was higher among girls and LM% among boys. Classifications of body composition in children are needed.

**Supplementary Information:**

The online version contains supplementary material available at 10.1186/s12887-026-06654-4.

## Introduction

The nutritional assessment of children is essential for health, especially due to the observed worldwide increase in overweight and obesity during childhood [1, 2]. Typically, this assessment is carried out by measuring weight and height and, subsequently, classifying individuals according to nutritional indicators, considering age [3]. However, these measures cannot differentiate lean mass from fat mass [4], making the assessment of body composition difficult, especially of fat mass, as it is an objective measure that can predict future health problems [5]. Also, body composition has been associated with physical activity, sleep, consumption of ultra-processed foods, and lung function [6–9].

The increase in overweight and obesity has become a global health problem [1]. In children and adolescents aged 5–19 worldwide, the prevalence of overweight (including obesity) is 20% [10]. This increase should be analyzed in detail, considering that excessive weight gain in childhood and adolescence is associated with premature death in adulthood [11], especially due to cardiovascular diseases [12]. Some studies have shown that being overweight in childhood is strongly associated with being overweight later in life [13]. Also, there is evidence that high Body Mass Index (BMI) in childhood may be associated with fat mass in adolescence and adulthood [13] and, consequently, with several chronic diseases, such as diabetes and high blood pressure [12].

In addition to evaluating child growth indicators using BMI, measuring body composition in children is essential to differentiate lean from fat mass [3–5]. Nutritional assessment is usually carried out using anthropometric measurements, but it is recognized that BMI, for example, alone doesn’t provide a deeper understanding of body composition. Still, these measures do not assess body composition, and it is necessary to use equipment such as air displacement plethysmography (BodPod) or Dual-Energy X-ray Absorptiometry (DEXA) [14, 15], even though other methods are also reported in the literature [16]. In Brazil, data on children’s body composition are scarce [15]. Given the nutritional transitions in the country and the ongoing increase in childhood obesity, it is necessary to assess children’s body composition [2, 17]. These data can contribute to the literature and the development of population policies.

This study aims to describe body composition (fat and lean mass) in children aged 6–7 from the 2015 Pelotas (Brazil) Birth Cohort.

## Methods

### Sample and ethical aspects

This article used data from the 2015 Pelotas (Brazil) Birth Cohort. From January 1 st to December 31 st, 2015, children born in the hospitals of the urban area of Pelotas, a mid-sized city in Southern Brazil, were eligible to participate in the Cohort. The number of eligible live births was 4,333, with 4,275 comprising the original Cohort sample. In the perinatal study, on the same occasion as delivery, mothers who responded to the questionnaire provided information on prenatal care, socioeconomic and demographic characteristics, physical activity, and other topics. The participants were invited to further follow-up assessments at 3 and 12 months, and at 2, 4, and 6–7 years. Of the 4,275 children included in the original 2015 birth cohort 97.2% (*N* = 4,110) were followed at three months, 95.4% (*N* = 4,018) at 1 year, 95.4% (*N* = 4,014) at 2 years, 95.4% (*N* = 4,010) at 4 years and 92.0% (*N* = 3,867) at 6–7 years for age [18, 19]. In this study, the analytical sample included children with valid body composition measurements who attended a visit to the study clinic with their mothers/guardians.

Maternal and child health variables were evaluated since birth, with repeated follow-ups at 3, 12 months, and 2, 4, and 6–7 years. Between November 2021 and November 2022, children were measured (6-7y follow-up) to evaluate several aspects of health, such as nutritional status, physical activity, screen time, COVID-19 antibodies, mental health, and body composition, among others. Of the 3,867 children followed, 76% attended the study clinic visit, where body composition equipment is located.

The 2015 Pelotas Birth Cohort study was approved by the School of Physical Education Ethics Committee at the Federal University of Pelotas (Registration number: 26746414.5.0000.5313) until the 4-year follow-up, and the 6–7-year follow-up was approved by the Faculty of Medicine Committee at the Federal University of Pelotas (Registration number: 51789921.1.0000.5317). Written informed consent was obtained from children’s parents or legal guardians and children were free to choose if they wanted to be evaluated in all tests and measures. Additional information on the logistics of the 2015 Pelotas (Brazil) Birth Cohort Study has been published elsewhere [18, 19].

### Body composition and BMI-for-age indicator

Body composition was evaluated using two objective measures – (1) Dual-Energy X-ray Absorptiometry (DEXA) and (2) Air-Displacement Plethysmography (BodPod). Both measurements were conducted by a trained technician, with the children wearing light, fitted clothing (shorts and sleeveless shirts) and no accessories. No exclusion criteria were adopted for these measures. Fat mass (FM) in kilograms (kg) and percentage (FM%) and lean mass (LM) in kilograms (kg) and percentage (LM%) and total body mass (TBM) in kilograms were obtained using a DEXA with EnCORE software platform (Lunar Prodigy, GE HealthCare, USA) and BodPod that provides a direct estimation of body composition. Weight was measured with a TANITA^®^ UM-080 scale, with a maximum capacity of 150 kg and 100 g precision. Height was measured at the clinic using a stadiometer with a precision of 1 mm and a maximum capacity of 2.06 m (Harpenden, Holtain, Crymych, UK) and at home using a stadiometer with a precision of 1 mm and a maximum capacity of 2.13 m (Alturexata, Belo Horizonte, Brazil). BMI-for-age in z-scores was calculated according to the World Health Organization Growth Standards, using Anthro 2005 software [3, 20]. The anthropometrists were trained and standardized for these measurements.

### Covariables

Socioeconomic and demographic variables were used as correlates of body composition results. Variables from the perinatal study were: child’s sex (female, male), maternal age at delivery in years (< 20, 20–34 and 35+), maternal skin color (white, brown, black), maternal education in years (0–4, 5–8, 9–11 and 12+), family income (quintiles), prepregnancy BMI (< 25.0, 25.0–30.0, ≥ 30.0 kg/m^2^), gestational hypertension (yes, no), gestational diabetes (yes, no), parity (1, 2, 3 or more) and prematurity (yes, no).

### Statistical analysis

Body composition was described using mean, standard deviation, and 95% confidence intervals (95%CI). The same approach was used to consider the independent variables and anthropometric indicators (weight-for-age, height-for-age, and BMI-for-age in z-scores). To test any differences in body composition according to the sample characteristics, Students’ t-tests were used for binary variables, and ANOVA was used for categorical and ordinal variables. All analyses were run using STATA 15.0 and considered a *p*-value of 0.05.

## Results

Of the 4,275 children participating in the original cohort. At 6–7 years, 3,867 were followed, 2,181 presented data for BodPod, and 2,271 for DEXA. At birth, most were born to women between 20 and 34 years of age (70.6%), and 31.1% of the mothers had 12 years or more of schooling. More than two-thirds of the mothers reported having white skin color. Most mothers (53.0%) had a pre-pregnancy BMI < 25.0 kg/m^2^. Only 25.5% and 8.6% of the women reported having hypertension and gestational diabetes, respectively. As for gestational age, 15.5% of the children were premature, 49.8% were first children, and 50.6% were male (Table 1). For the BodPod sample, slight differences were observed for skin color, schooling, income, pre-pregnancy BMI, and prematurity, and DEXA for skin color, schooling, pre-pregnancy BMI, parity, and prematurity (Table 1).

**Table 1:** Sample characteristics of the original cohort and according to equipment. 2015 Birth Cohort, Pelotas/RS, Brazil

	Original cohort (*N* = 4,275)		BodPod (*N* = 2,181)		DXA (*N* = 2,271)		
	*N* (%)	95%CI	*N* (%)	95%CI	*N* (%)		95%CI
Maternal age at delivery (in years)							
< 20	623 (14.6)	13.5; 15.7	291 (13.3)	12.0; 14.8	307 (13.5)		12.2; 15.0
20–34	3,018 (70.6)	69.2; 72.0	1,555 (71.3)	69.4; 73.2	1,621 (71.4)		69.5; 73.2
35+	633 (14.8)	13.8; 15.9	355 (15.4)	13.9; 16.9	343 (15.1)		13.7; 16.6
Maternal skin color							
White	3,024 (71.3)	69.9; 72.6	1,539 (71.0)	69.0; 72.8	1,599 (70.8)		68.9; 72.6
Brown	551 (13.0)	12.0; 16.9	259 (11.9)	10.6; 13.4	271 (12.0)		10.7; 13.4
Black	667 (15.7)	14.6; 16.8	371 (17.1)	15.6; 18.7	389 (17.2)		15.7; 18.8
Maternal education (years)							
0–4	391 (9.2)	8.3; 10.0	171 (7.8)	6.8; 9.0	174 (7.7)		6.6; 8.8
5–8	1,095 (25.6)	24.3; 26.9	492 (22.6)	20.8; 24.4	520 (22.9)		21.2; 24.7
9–11	1,458 (34.1)	32.7; 35.5	822 (37.7)	35.7; 39.7	850 (37.4)		35.4; 39.4
12+	1,330 (31.1)	29.7; 32.5	696 (31.9)	30.0; 33.9	727 (32.0)		30.1; 34.0
Family income (in quintiles)							
1st (poorest)	846 (19.8)	18.6; 21.0	398 (18.3)	16.7; 19.9	419 (18.4)		16.9; 20.1
2nd	859 (20.1)	18.9; 21.3	449 (20.6)	18.9; 22.3	456 (20.1)		18.5; 21.8
3rd	853 (20.0)	18.8; 21.2	451 (20.7)	19.0; 22.4	467 (20.6)		19.0; 22.3
4th	856 (20.0)	18.8; 21.3	458 (21.0)	19.3; 22.8	482 (21.2)		19.6; 23.0
5th (richest)	859 (20.1)	18.9; 21.3	424 (19.4)	17.8; 21.2	446 (19.7)		18.1; 21.3
Pre-pregnancy BMI (kg/m^2^)							
< 25.0	2,193 (53.0)	51.4; 54.5	1,075 (50.8)	48.6; 52.9	1,125 (51.0)		48.9; 53.0
25.0 until < 30.0	1,169 (28.2)	26.9; 29.6	619 (29.2)	27.3; 31.2	643 (29.1)		27.3; 31.0
≥ 30.0	779 (18.8)	17.6; 20.0	424 (20.0)	18.4; 21.8	440 (19.9)		18.3; 21.6
Gestational arterial hypertension							
No	3,183 (74.5)	73.2; 75.8	1,619 (74.3)	72.4; 76.0	1,689 (74.4)		72.6; 76.2
Yes	1,089 (25.5)	24.2; 26.8	561 (25.7)	23.9; 27.6	581 (25.6)		23.8; 27.4
Gestational diabetes							
No	3,906 (91.4)	90.5; 92.2	1,979 (90.7)	89.4; 91.9	2,067 (91.0)		89.8; 92.1
Yes	366 (8.6)	7.8; 9.4	202 (9.3)	8.1; 10.6	204 (9.0)		7.9; 10.2
Parity (number of previous children)							
1	2,112 (49.4)	47.9; 50.9	1,100 (50.4)	48.3; 52.5	1,156 (50.9)		48.8; 53.0
2	1,321 (30.9)	29.5; 32.3	675 (91.0)	29.0; 32.9	700 (30.8)		29.0; 32.7
3 or more	840 (19.7)	18.5; 20.9	406 (18.6)	17.0; 20.3	415 (18.3)		16.7; 19.9
Prematurity							
Yes	663 (15.5)	14.4; 16.6	307 (14.1)	12.7; 15.6	316 (13.9)		12.5; 15.4
No	3,612 (84.5)	83.4; 85.5	1,874 (85.9)	84.4; 87.3	1,955 (86.1)		84.6; 87.4
Child sex							
Female	2,111 (49.4)	47.9; 50.9	1,081 (49.6)	48.3; 52.5	1,144 (50.4)		47.6; 51.7
Male	2,164 (50.6)	49.1; 52.1	1,100 (50.4)	47.5; 51.7	1,127 (49.6)		48.3; 52.4

*95%CI* Confidence interval

**Fig. 1:**
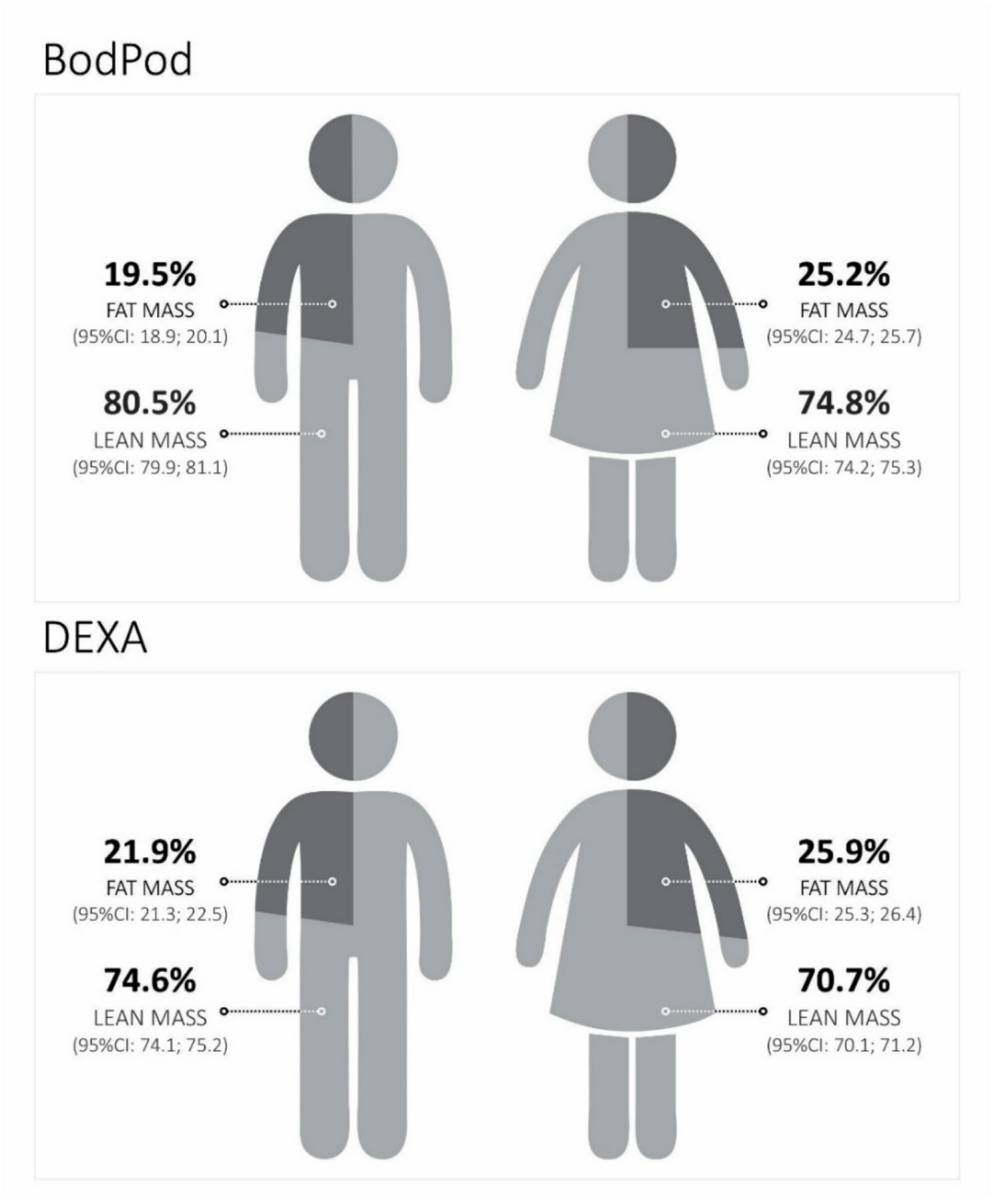
Mean fat mass percentage (FM%) and lean mass percentage (LM%) by equipment and sex among children aged 6–7 years from the 2015 Birth Cohort, Pelotas/RS, Brazil

Overall, girls presented more fat mass on average, while boys presented more lean mass. In the DEXA, the mean FM% among girls was 25.9% (95%CI 25.3; 26.4), and among boys, 21.9% (95%CI 21.3; 22.5), and in the BodPod was 25.2% (95%CI 24.7; 25.7) among girls and 19.5% (95%CI 18.9; 20.1) for the boys (Fig. 1). On average, anthropometric indicators also differed between girls and boys, except for the height-for-age indicator (Table 2). The average z-scores for weight-for-age and BMI-for-age were higher in boys than in girls, with values of 0.97 (95% CI 0.90; 1.04) and 0.71 (95% CI 0.65; 0.78), respectively (Table 2).

**Table 2:** Mean of body composition measures and z-scores of anthropometric indicators, overall and by sex. 2015 Birth Cohort, Pelotas/RS, Brazil

	Overall			Girls			Boys			*p*-value
	Mean (SD)	Min	Max	Mean (SD)	Min	Max	Mean (SD)	Min	Max	
DEXA										
FM (kg)	6.7 (4.7)	6.5	6.9	7.02 (4.4)	6.8	7.3	6.3 (4.9)	6.0	6.6	< 0.001
FM (%)	23.9 (10.2)	23.5	24.3	25.9 (9.8)	25.3	26.4	21.9 (10.3)	21.3	22.5	< 0.001
LM (kg)	18.0 (5.7)	17.9	18.1	17.2 (2.5)	17.1	17.3	18.8 (2.7)	18.3	18.9	< 0.001
LM (%)	72.7 (20.9)	72.2	73.1	70.7 (9.5)	70.1	71.2	74.6 (10.0)	74.1	75.2	< 0.001
TBM (kg)	25.5 (14.1)	25.2	25.8	25.1 (6.4)	24.7	25.4	25.9 (7.0)	25.5	26.4	0.002
BodPod										
FM (kg)	6.5 (5.3)	6.3	6.7	7.2 (5.6)	6.8	7.5	5.8 (4.9)	5.5	6.1	< 0.001
FM (%)	22.3 (21.5)	21.9	22.7	25.2 (8.9)	24.7	25.7	19.5 (10.2)	18.9	20.1	< 0.001
LM (kg)	19.7 (3.0)	19.6	19.9	18.7 (2.8)	18.6	18.9	20.7 (2.9)	20.6	20.9	< 0.001
LM (%)	77.7 (10.0)	77.3	78.1	74.8 (9.0)	74.2	75.3	80.5 (10.2)	79.9	81.1	< 0.001
TBM (kg)	26.0 (6.6)	25.8	26.3	25.6 (6.3)	25.3	26.0	26.5 (6.9)	26.1	26.9	0.003
Anthropometrics indicators										
Weight-for-age z-score	0.88 (1.4)	0.84	0.93	0.79 (1.3)	0.72	0.85	0.97 (1.4)	0.91	1.04	< 0.001
Height-for-age z-score	0.48 (1.1)	0.45	0.52	0.46 (1.1)	0.41	0.51	0.50 (1.1)	0.45	0.55	0.267
BMI-for-age z-score	0.84 (1.4)	0.79	0.89	0.71 (1.4)	0.65	0.78	0.97 (1.5)	0.90	1.04	< 0.001

*FM (kg)* fat mass in kilogram, *FM (%) *fat mass in percentage, *LM (kg) *lean mass in kilogram, *LM (%) *lean mass in percentage, *TBM (kg) *total body mass in kilogram, *SD* Standard Deviation

It was observed that the highest mean FM% and FM in kilograms (DEXA and BodPod) were observed in children whose mothers were over 35 years old, had higher levels of education, higher pre-pregnancy BMI, hypertension, and diabetes during pregnancy, and who belonged to the third- and fourth-intermediate income quintiles. Children of mothers with more kids showed lower fat mass scores (Table 3). A statistically significant association was found for lean mass with the same variables, but in the opposite direction. However, for the parity variable, children whose mothers had more children had higher LM% and LM in kilograms (Table 3). The same associations were observed according to sex (Supplementary Tables 1 and 2).

**Table 3:** Mean of body composition measures according to independent variables. 2015 Birth Cohort, Pelotas/RS, Brazil

	DEXA					BodPod				
	FM		LM		TBM	FM		LM		TBM
	%	Kg	%	Kg	Kg	%	Kg	%	Kg	Kg
	**β ** **(95%CI)**	**β ** **(95%CI)**	**β ** **(95%CI)**	**β ** **(95%CI)**	**β ** **(95%CI)**	**β ** **(95%CI)**	**β ** **(95%CI)**	**β ** **(95%CI)**	**β ** **(95%CI)**	**β ** **(95%CI)**
Maternal age at deliver (in years)										
< 20	22.9 (21.9; 24.0)	6.1 (5.7 6.5)	73.6 (72.6; 74.6)	17.8 (17.5; 18.1)	24.7 (24.0; 25.4)	21.0 (20.0; 22.1)	5.7 (5.2; 6.1)	79.0 (77.9; 80.0)	19.5 (19.2; 19.8)	25.2 (24.5; 25.8)
20–34	23.8 (23.3, 24.3)	6.7 (6.4; 6.9)	72.7 (72.2; 73.2)	18.0 (17.9; 18.1)	25.5 (25.2; 25.8)	22.3 (21.8; 22.8)	6.5 (6.3; 6.8)	77.7 (77.2; 78.2)	19.7 (19.6; 19.9)	26.1 (25.7; 26.4)
35+	25.2 (24.0; 26.3)	7.2 (6.7; 7.7)	71.4 (70.4; 72.5)	18.2 (17.9; 18.4)	26.3 (25.5; 27.0)	23.3 (22.2; 24.4)	7.0 (6.3; 7.7)	76.6 (75.5; 77.7)	20.0 (19.6; 20.3)	26.8 (26.0; 27.5)
Maternal skin color										
White	24.2 (23.7; 24.7)	6.7 (6.5; 6.9)	72.4 (71.9; 72.9)	18.0 (17.8; 18.1)	25.5 (25.2; 25.9)	22.7 (22.3; 23.2)	6.6 (6.3; 6.8)	77.2 (76.8; 77.7)	19.7 (19.6; 19.9)	26.1 (25.8; 26.4)
Brown	23.4 (22.3; 24.6)	6.7 (6.2; 7.3)	73.0 (72.0; 74.1)	18.1 (17.8; 18.4)	25.7 (24.9; 26.5)	21.3 (20.2; 22.4)	6.2 (5.7; 6.7)	78.7 (77.6; 79.8)	19.9 (19.6; 20.2)	26.0 (25.3; 26.8)
Black	22.9 (21.7; 24.2)	6.3 (5.8; 6.9)	73.6 (72.4; 74.8)	18.0 (17.7; 18.3)	25.2 (24.4; 25.9)	21.3 (20.0; 22.5)	6.5 (5.6; 7.3)	78.7 (77.5; 80.0)	19.8 (19.4; 20.1)	25.8 (25.0; 26.6)
Maternal education (years)										
0–4	21.3 (19.7; 22.9)	5.8 (5.1; 6.6)	75.2 (73.7; 76.7)	18.0 (17.6; 18.3)	24.6 (23.6; 25.7)	19.7 (18.2; 21.3)	5.5 (4.8; 6.3)	80.3 (78.7; 81.8)	19.6 (19.1; 20.1)	25.1 (24.0; 26.2)
5–8	22.7 (21.8; 23.5)	6.1 (5.8; 6.5)	73.8 (73.0; 74.6)	17.8 (17.6; 18.0)	24.8 (24.2; 25.3)	21.2 (20.3; 22.0)	6.0 (5.5; 6.4)	78.8 (77.9; 79.7)	19.6 (19.4; 19.9)	25.5 (24.9; 26.0)
9–11	24.7 (24.0; 25.4)	6.9 (6.6; 7.3)	71.9 (71.2; 72.6)	18.0 (17.8; 18.2)	25.8 (25.4; 26.3)	23.0 (22.3; 23.7)	6.8 (6.4; 7.2)	77.0 (76.3; 77.7)	19.7 (19.5; 20.0)	26.3 (25.8; 26.8)
12+	24.4 (23.7; 25.2)	6.9 (6.5; 7.2)	72.1 (71.4; 72.9)	18.1 (17.9; 18.3)	25.9 (25.4; 26.4)	23.0 (22.2; 23.7)	6.7 (6.3; 7.1)	77.1 (76.3; 77.8)	19.9 (19.6; 20.1)	26.4 (25.9; 26.9)
Family income (in quintiles)										
1 st (poorest)	22.0 (21.1; 23.0)	5.9 (5.5; 6.3)	74.4 (73.5; 75.3)	17.7 (17.4; 18.0)	24.4 (23.8; 25.1)	20.8 (19.9; 21.8)	5.9 (5.3; 6.5)	79.1 (78.2; 80.1)	19.5 (19.2; 19.8)	25.2 (24.6; 25.9)
2nd	23.0 (22.1; 24.0)	6.3 (5.9; 6.7)	73.5 (72.6; 74.4)	17.9 (17.6; 18.1)	25.0 (24.4; 25.6)	21.8 (20.9; 22.8)	6.1 (5.7; 6.5)	78.2 (77.2; 79.1)	19.5 (19.2; 19.8)	25.6 (25.0; 26.2)
3rd	25.2 (24.2; 26.1)	7.2 (6.8; 7.7)	71.4 (70.5; 72.3)	18.2 (17.9; 18.5)	26.3 (25.7; 27.0)	23.3 (22.4; 24.3)	7.3 (6.6; 7.9)	76.7 (75.7; 77.6)	20.0 (19.7; 20.3)	26.9 (26.2; 27.6)
4th	25.4 (24.4; 26.3)	7.3 (6.8; 7.7)	71.2 (70.3; 72.1)	18.1 (17.9; 18.3)	26.2 (25.6; 26.8)	23.6 (22.7; 24.6)	6.8 (6.4; 7.2)	76.4 (75.5; 77.4)	19.8 (19.6; 20.1)	26.6 (26.0; 27.3)
5th (richest)	23.6 (22.7; 24.4)	6.4 (6.1; 6.8)	73.0 (72.1; 73.8)	18.1 (17.9; 18.3)	25.4 (24.8; 25.9)	21.7 (20.8; 22.6)	6.3 (5.8; 6.7)	78.3 (77.4; 79.2)	19.8 (19.6; 20.1)	25.8 (25.3; 26.3)
Pre-pregnancy BMI (kg/m^2^)										
< 25.0	21.7 (21.2; 22.3)	5.6 (5.4; 5.8)	74.7 (74.2; 75.3)	17.6 (17.4; 17.7)	24.0 (23.7; 24.3)	20.6 (20.0; 21.1)	5.5 (5.3; 5.8)	79.4 (78.8; 80.0)	19.2 (19.0; 19.3)	24.6 (24.3; 24.9)
25.0 until < 30.0	24.6 (23.8; 25.4)	6.9 (6.6; 7.3)	72.0 (71.2; 72.8)	18.2 (18.0; 18.4)	26.0 (25.5; 26.5)	22.8 (22.0; 23.6)	6.8 (6.3; 7.3)	77.3 (76.4; 78.1)	20.0 (19.8; 20.2)	26.5 (26.0; 27.0)
≥ 30.0	28.6 (27.6; 29.6)	8.9 (8.4; 9.5)	68.1 (67.1; 69.1)	18.7 (18.4; 19.0)	28.6 (27.8; 29.3)	26.5 (25.5; 27.5)	8.5 (8.0; 9.1)	73.5 (72.5; 74.5)	20.8 (20.4; 21.1)	29.2 (28.4; 30.0)
Gestational arterial hypertension										
No	23.2 (22.7; 23.6)	6.3 (6.1; 6.5)	73.3 (72.9; 73.8)	17.9 (17.7; 18.0)	25.1 (24.8; 25.4)	21.7 (21.2; 22.2)	6.2 (6.0; 6.5)	78.3 (77.8; 78.8)	19.6 (19.4; 19.7)	25.6 (25.3; 25.9)
Yes	26.0 (25.1; 26.8)	7.6 (7.2; 8.1)	70.7 (69.8; 71.5)	18.3 (18.1; 18.5)	26.8 (26.2; 27.4)	24.1 (23.2; 25.0)	7.2 (6.8; 7.6)	75.9 (75.0; 76.8)	20.2 (19.9; 20.5)	27.4 (26.8; 28.0)
Gestational diabetes										
No	23.5 (23.1; 23.9)	6.5 (6.3; 6.6)	73.0 (72.6; 73.5)	17.9 (17.8; 18.0)	25.2 (24.9; 25.5)	22.0 (21.5; 22.4)	6.3 (6.1; 6.5)	78.0 (77.6; 78.4)	19.6 (19.5; 19.8)	25.8 (25.5; 26.0)
Yes	27.9 (26.3; 29.5)	8.7 (7.9; 9.6)	68.7 (67.2; 70.3)	18.7 (18.3; 19.1)	28.4 (27.2; 29.5)	25.6 (24.1; 27.1)	8.3 (7.4; 9.1)	74.4 (72.9; 75.9)	20.7 (20.3; 21.2)	28.8 (27.7; 30.0)
Parity (number of previous children)										
1	24.5 (24.0; 25.1)	6.9 (6.6; 7.2)	72.0 (71.4; 72.6)	18.0 (17.9; 18.2)	25.8 (25.4; 26.2)	22.7 (22.1; 23.3)	6.6 (6.3; 6.9)	77.3 (76.7; 77.9)	19.8 (19.6; 20.1)	26.3 (25.9; 26.7)
2	23.6 (22.8; 24.3)	6.5 (6.2; 6.9)	73.0 (72.2; 73.7)	17.9 (17.6; 18.0)	25.2 (24.7; 25.7)	22.5 (21.7; 23.2)	6.6 (6.2; 7.1)	77.5 (76.8; 78.3)	19.6 (19.4; 19.9)	26.0 (25.5; 26.5)
3 or more	22.6 (21.6; 23.6)	6.2 (5.8; 6.7)	73.9 (72.9; 74.9)	18.0 (17.8; 18.3)	25.1 (24.5; 25.8)	21.0 (20.0; 22.0)	5.9 (5.4; 6.3)	79.0 (78.0; 80.0)	19.7 (19,4; 20.0)	25.6 (24.9; 26.2)
Prematurity										
No	24.1 (23.6; 24.6)	6.8 (6.6; 7.0)	72.4 (72.0; 72.9)	18.0 (17.9; 18.2)	25.7 (25.4; 26.0)	22.6 (22.1; 23.1)	6.6 (6.4; 6.9)	77.4 (76.9; 77.9)	19.6 (19.4; 19.9)	26.2 (25.9; 26.5)
Yes	22.6 (21.5; 23.7)	5.9 (5.5; 6.4)	73.9 (72.9; 74.9)	17.7 (17.4; 17.9)	24.5 (23.9; 25.1)	20.6 (19.5; 21.6)	5.6 (5.1; 6.0)	79.4 (78.3; 80.5)	19.7 (19.4; 20.0)	25.0 (24.4; 25.6)

*FM (kg) *fat mass in kilogram, *FM (%) *fat mass in percentage, *LM (kg) *lean mass in kilogram, *LM (%) *lean mass in percentage, *TBM (kg) *total body mass in kilogram, *SD *Standard Deviation, *95%CI* Confidence interval

Table 4 shows the classification of standardized BMI-for-age by FM%. Children classified as obese at 6–7 years of age had a higher average FM% for both DEXA (37.2%) and BodPod (34.5%). Girls presented a higher percentage of fat mass than boys, particularly among those with obesity, where the percentage was 39.6% (DEXA) and 37.4% (BodPod). There were no statistically significant differences in the mean anthropometric indicators between children with body composition measurements and those without (Table Supplementary 3).

**Table 4:** Mean fat mass percentage (FM%) measured using DEXA and BodPod according to BMI-for-age z-score, overall and by sex. 2015 Birth Cohort, Pelotas/RS, Brazil

BMI-for-age z-score	Overall		Girls		Boys	
	Mean (SD)	95%CI	Mean (SD)	95%CI	Mean (SD)	95%CI
DEXA						
Low-normal	17.5 (5.7)	17.2; 17.8	19.9 (5.5)	19.4; 20.3	14.9 (4.7)	14.5; 15.2
Overweight	27.3 (6.0)	26.7; 27.8	30.5 (5.3)	29.8; 31.2	24.2 (5.0)	23.6; 24.9
Obesity	37.2 (6.2)	36.6; 37.7	39.6 (5.5)	38.9; 40.3	34.8 (5.9)	34.1; 35.6
BodPod						
Low-normal	16.6 (6.5)	16.3; 17.0	20.0 (5.6)	19.6; 20.4	13.0 (5.3)	12.5; 13.4
Overweight	24.9 (6.8)	24.2; 25.5	29.1 (5.3)	28.3; 29.8	21.2 (5.8)	20.4; 21.9
Obesity	34.5 (6.4)	33.9; 35.1	37.4 (5.2)	36.7; 38.1	31.8 (6.3)	31.0; 32.6

*SD* Standard Deviation, *95%CI *Confidence interval

## Discussion

The mean fat mass percentage was 23.9% in the DEXA and 22.3% in the BodPod, showing a difference of 1.6% points. Lean mass percentage was 72.7% (DEXA) and 77.7% (BodPod), with a greater difference between the two equipment. The main explanation for this difference could be that DEXA evaluates tissues individually, while the BodPod uses air displacement, which would lead to overestimation of lean mass and underestimation of fat mass [14–16].

Among girls, the mean fat mass percentage was higher in both methods, while the mean lean mass percentage was higher among boys. Some differences were identified in body composition indicators according to the independent variables, suggesting that higher mean FM% can be attributed to children from families with better socioeconomic conditions. By evaluating the anthropometric indicators of weight-for-age, height-for-age, and BMI-for-age in z-score, it was possible to identify that those children classified as obese by the BMI-for-age indicator in z-score had a higher mean FM%.

Although the literature shows that differences related to body composition become more pronounced at puberty [21], it is known that there are differences in the distribution of fat mass and lean mass between girls and boys [22]. The magnitude of these differences may be more pronounced in the maturation phase and from the end of puberty to the beginning of adulthood [23]; however, subtle differences have been observed earlier.

In this study, the differences found, according to sex were consistent with the literature. A study conducted in southeastern Poland with schoolchildren aged 7 to 13 years, which assessed body composition using DEXA, identified that girls had a higher percentage of fat mass, while boys had a higher percentage of lean mass [24]. This was also evidenced in another cohort study conducted in Pelotas-RS, Brazil, within the same age group that measured body composition using DEXA. The authors found 23.4% of fat mass percentage, which was also higher among girls [15].

The mechanisms that influence the distribution of body fat are not well understood, but some changes that occur throughout the life cycle can be explained by hormonal variations that tend to be more pronounced in women [25]. However, fat distribution also depends on age, with men more likely to store visceral fat, while women tend to accumulate subcutaneous fat. As age increases, men and women accumulate more visceral fat [26].

There is little evidence to assess the body composition of children at specific ages, such as 6–7 years old, as most studies focus on broader age groups, such as children and adolescents or schoolchildren [14–16, 24]. Still, no classification can be used as a reference for low or high fat mass percentages. In the present study, an analysis was conducted to determine the mean FM% for each BMI-for-age classification category in z-scores. The results showed that children classified as overweight or obese based on BMI-for-age in z-scores had a higher mean FM%. The correlation between BMI-for-age and FM% was strong (*R* = 0.84 – data not shown). According to evidence, body composition-related disorders are multicausal, such as those associated with anthropometric indicators [27]. These factors can be genetic, environmental, and socioeconomic [24, 28, 29]. This is supported by the findings of this study, which showed that income, maternal education, pre-pregnancy BMI, diabetes, and gestational hypertension were associated with higher mean body fat.

The results of this study showed a significant association between pre-pregnancy maternal BMI and body fat in children, especially girls whose mothers had a BMI ≥ 30.0 kg/m2 before pregnancy, and a higher mean fat mass. This finding is consistent with the literature, suggesting that pre-pregnancy maternal BMI, especially overweight, is a strong predictor of weight gain and an increased BMI in offspring during childhood and throughout life [30, 31], also impacting body composition. A meta-analysis of 23 studies showed a significant association between parents and children with overweight and obesity [28]. This relationship was stronger when parents and children were overweight [28], suggesting an intergenerational influence.

A higher average FM% was observed in children whose mothers had a higher level of education and who belonged to families with higher incomes. Other studies have also found this association [1, 2, 24], which may be influenced by these factors affecting individual choices early in life, increasing the occurrence of overweight and obesity in the medium and long term [8, 32]. The literature suggests that diet and physical activity can influence the association between body fat and variables such as education and income [33]. However, further analytical approaches are needed to confirm these hypotheses.

A potential limitation of this study is that the sample evaluated using DEXA and BodPod was smaller than the sample assessed in the 6–7-year follow-up of the birth cohort. This was due to logistical challenges faced during the COVID-19 pandemic. Several efforts were made to maximize measures related to body composition, including weekend schedules, given that 76% of participants attended the clinic study visit. However, sensitivity analyses showed no statistically significant differences in the means of the anthropometric indicators of weight-for-age, height-for-age, and BMI-for-age in z-scores when comparing children who did and did not take the DEXA and BodPod assessments.

## Conclusion

The average FM% was higher among girls and those children classified as obese according to the anthropometric indicator of BMI-for-age z-score. The findings of this study can be used for the development of future public policies, particularly in creating classifications that consider these differences, enabling preventive actions since body composition can predict future health problems. Based on these findings, further studies can explore body composition to verify associations that may also contribute to developing strategies and classifications, facilitating a comprehensive assessment of children’s health up to the beginning of adolescence. This is an important finding as the FM% should be monitored in all ages to prevent future issues, such as chronic diseases. It is necessary to develop classifications similar to those used for BMI, for example, such as those proposed by the World Health Organization [20], to classify children based on body composition measurements.

## Supplementary Information

Supplementary Material 1.

## Data Availability

The datasets used and/or analyzed during the current study are available from the corresponding author on reasonable request.

## Abbreviations

FM: Fat mass
LM: Lean mass
TBM: Total body mass
DEXA: Dual-Energy X-ray Absorptiometry
BodPod: Air-Displacement Plethysmography
BMI: Body mass index
