# Supplementary material for: Body composition among 6–7 years-old children: data from the 2015 Pelotas (Brazil) Birth Cohort Study
Source: BMC Pediatr. 2026 Mar 5;26:297. doi: 10.1186/s12887-026-06654-4 (PMC13069724; doi:10.1186/s12887-026-06654-4)
Supplement: Supplementary file 1 — Supplementary Material 1. [file 12887_2026_6654_MOESM1_ESM.docx]

Supplementary material

**Supplementary Table 1.** Mean of body composition measures according to independent variables in girls. 2015 Birth Cohort, Pelotas/RS, Brazil.

|  | **DEXA** | | | | | **BodPod** | | | | |
| --- | --- | --- | --- | --- | --- | --- | --- | --- | --- | --- |
|  | **FM** |  | **LM** |  | **TBM** | **FM** |  | **LM** |  | **TBM** |
|  | **%** | **Kg** | **%** | **Kg** | **Kg** | **%** | **Kg** | **%** | **Kg** | **Kg** |
|  | **β**  **(95%CI)** | **β**  **(95%CI)** | **β**  **(95%CI)** | **β**  **(95%CI)** | **β**  **(95%CI)** | **β**  **(95%CI)** | **β**  **(95%CI)** | **β**  **(95%CI)** | **β**  **(95%CI)** | **β**  **(95%CI)** |
| **Maternal age at delivery**  **(in years)** |  |  |  |  |  |  |  |  |  |  |
| < 20 | 24.9  (23.6; 26.2) | 6.4  (5.8; 6.9) | 71.6  (70.3; 72.8) | 17.0  (16.6; 17.3) | 24.2  (23.3; 25.0) | 24.2  (22.9; 26.1) | 6.4  (5.8; 6.9) | 75.8  (74.5; 77.1) | 18.5  (18.1; 18.9) | 24.9  (24.0; 25.7) |
| 20-34 | 26.1  (25.4, 26.8) | 7.2  (6.8; 7.5) | 70.4  (69.8; 71.1) | 17.2  (17.1; 17.4) | 25.2  (24.8; 25.7) | 25.5  (24.9; 26.1) | 7.3  (6.9; 7.7) | 74.5  (73.8; 75.1) | 18.8  (18.6; 19.0) | 25.9  (25.4; 26.3) |
| 35+ | 25.6  (24.0; 27.2) | 7.0  (6.3; 7.7) | 71.0  (69.4; 72.5) | 17.2  (16.8; 17.5) | 25.0  (24.0; 25.9) | 24.7  (23.2; 26.2) | 7.1  (6.0; 8.3) | 75.2  (73.8; 76.7) | 18.4  (18.1; 18.9) | 25.2  (24.2; 26.1) |
| **Maternal skin color** |  |  |  |  |  |  |  |  |  |  |
| White | 26.0  (25.4; 26.7) | 7.0  (6.7; 7.3) | 70.5  (69.9; 71.2) | 17.2  (17.0; 17.3) | 25.0  (24.6; 25.5) | 25.7  (25.0; 26.3) | 7.2  (6.9; 7.6) | 74.3  (73.7; 74.9) | 18.6  (18.4; 18.8) | 25.7  (25.2; 26.1) |
| Brown | 25.5  (24.0; 27.0) | 7.1  (6.4; 7.3) | 71.0  (69.6; 72.4) | 17.3  (16.9; 17.7) | 25.3  (24.3; 26.3) | 23.9  (22.6; 25.2) | 6.6  (6.0; 7.2) | 76.1  (74.8; 77.4) | 19.0  (18.6; 19.4) | 25.6  (24.7; 26.5) |
| Black | 25.6  (23.9; 27.3) | 6.9  (6.1; 7.8) | 70.9  (69.3; 72.6) | 17.2  (16.8; 17.6) | 25.0  (23.8; 26.1) | 24.4  (22.7; 26.1) | 7.8  (6.1; 9.4) | 75.6  (73.9; 77.2) | 18.9  (18.4; 19.3) | 25.6  (24.4; 26.8) |
| **Maternal education (years)** |  |  |  |  |  |  |  |  |  |  |
| 0-4 | 22.3  (20.2; 24.4) | 5.6  (4.7; 6.5) | 74.1  (72.1; 76.2) | 16.7  (16.3; 17.1) | 23.1  (21.9; 24.3) | 21.9  (19.9; 23.8) | 5.6  (4.7; 6.4) | 78.1  (76.1; 80.0) | 18.0  (17.5; 18.6) | 23.6  (22.3; 24.8) |
| 5-8 | 25.2  (24.0; 26.4) | 6.7  (6.1; 7.2) | 71.3  (70.2; 72.4) | 16.9  (16.6; 17.3) | 24.4  (23.6; 25.2) | 24.8  (23.7; 25.9) | 6.7  (6.2; 7.2) | 75.2  (74.1; 76.3) | 18.6  (18.2; 18.9) | 25.2  (24.5; 26.0) |
| 9-11 | 26.8  (25.9; 27.7) | 7.4  (7.0; 7.8) | 69.8  (68.9; 70.7) | 17.2  (17.0; 17.5) | 25.4  (24.8; 26.1) | 26.0  (25.1; 26.9) | 7.7  (7.0; 8.3) | 74.0  (73.1; 74.8) | 18.8  (18.5; 19.1) | 26.0  (25.4; 26.6) |
| 12+ | 26.0  (25.0; 27.0) | 7.2  (6.7; 7.6) | 70.5  (69.6; 71.5) | 17.5  (17.3; 17.7) | 25.5  (24.9; 26.1) | 25.3  (24.4; 26.3) | 7.3  (6.7; 7.8) | 74.6  (73.7; 75.6) | 18.9  (18.6; 19.2) | 25.9  (25.3; 26.6) |
| **Family income (in quintiles)** |  |  |  |  |  |  |  |  |  |  |
| 1^st^ (poorest) | 23.5  (22.2; 24.7) | 6.0  (5.4; 6.5) | 73.0  (71.7; 74.2) | 16.6  (16.2; 17.0) | 23.4  (22.5; 24.2) | 23.4  (22.2; 24.6) | 6.4  (5.5; 7.4) | 76.6  (75.4; 77.8) | 18.2  (17.9; 18.6) | 24.3  (23.5; 25.1) |
| 2^nd^ | 25.5  (24.2; 26.9) | 6.9  (6.3; 7.5) | 71.0  (69.7; 72.3) | 17.1  (16.8; 17.4) | 24.9  (24.0; 25.7) | 25.2  (23.9; 26.4) | 6.9  (6.3; 7.4) | 74.8  (73.6; 76.0) | 18.5  (18.2; 18.9) | 25.4  (24.6; 26.3) |
| 3^rd^ | 26.8  (25.5; 28.1) | 7.5  (6.8; 8.1) | 69.8  (68.6; 71.0) | 17.4  (17.1; 17.8) | 25.8  (24.9; 26.6) | 25.7  (24.5; 26.9) | 7.7  (6.8; 8.7) | 74.3  (73.1; 75.5) | 19.0  (18.6; 19.4) | 26.2  (25.3; 27.1) |
| 4^th^ | 27.7  (26.5; 29.0) | 7.8  (7.2; 8.4) | 68.9  (67.7; 70.1) | 17.3  (17.0; 17.7) | 26.0  (25.2; 26.9) | 27.2  (26.1; 28.3) | 7.7  (7.2; 8.3) | 72.8  (71.6; 73.9) | 18.9  (18.5; 19.3) | 26.6  (25.8; 27.5) |
| 5^th^ (richest) | 25.2  (24.0; 26.4) | 6.7  (6.2; 7.2) | 71.3  (70.1; 72.5) | 17.4  (17.1; 17.6) | 24.9  (24.2; 25.7) | 24.1  (22.9; 25.3) | 6.9  (6.1; 7.7) | 75.8  (74.7; 77.0) | 18.8  (18.5; 19.2) | 25.3  (24.6; 26.0) |
| **Pre-pregnancy BMI (kg/m^2^)** |  |  |  |  |  |  |  |  |  |  |
| < 25.0 | 24.0  (23.2; 24.7) | 6.1  (5.8; 6.4) | 72.5  (71.8; 73.2) | 16.9  (16.7; 17.1) | 23.9  (23.4; 24.3) | 23.8  (23.1; 24.5) | 6.3  (5.9; 6.6) | 76.2  (75.5; 76.9) | 18.3  (18.1; 18.5) | 24.5  (24.0; 24.9) |
| 25.0 until < 30.0 | 26.9  (25.8; 28.0) | 7.5  (7.0; 8.0) | 69.6  (68.6; 70.7) | 17.4  (17.1; 17.7) | 25.7  (25.0; 26.5) | 25.8  (24.8; 26.9) | 7.9  (7.0; 8.7) | 74.1  (73.1; 75.1) | 19.0  (18.7; 19.3) | 26.3  (25.6; 27.0) |
| ≥ 30.0 | 30.2  (28.8; 31.6) | 9.0  (8.3; 9.8) | 66.5  (65.1; 67.9) | 17.8  (17.4; 18.2) | 27.7  (26.7; 28.8) | 28.8  (27.5; 30.1) | 9.0  (8.1; 9.9) | 71.2  (69.9; 72.5) | 19.6  (19.1; 20.0) | 28.3  (27.2; 29.3) |
| **Gestational arterial**  **hypertension** |  |  |  |  |  |  |  |  |  |  |
| No | 25.2  (24.5; 25.8) | 6.7  (6.4; 7.0) | 71.4  (70.7; 72.0) | 17.1  (16.9; 17.2) | 24.6  (24.2; 25.0) | 24.6  (24.0; 25.2) | 7.0  (6.5; 7.4) | 75.4  (74.8; 76.0) | 18.6  (18.4; 18.8) | 25.2  (24.8; 25.6) |
| Yes | 28.0  (26.8; 29.1) | 8.0  (7.4; 8.6) | 68.7  (67.5; 69.8) | 17.5  (17.2; 17.9) | 26.4  (25.6; 27.3) | 27.1  (25.9; 28.2) | 7.8  (7.3; 8.4) | 72.9  (71.8; 74.0) | 19.1  (18.7; 19.4) | 26.9  (26.0; 27.8) |
| **Gestational diabetes** |  |  |  |  |  |  |  |  |  |  |
| No | 25.5  (24.9; 26.1) | 6.8  (6.6; 7.1) | 71.0  (70.5; 71.6) | 17.1  (17.0; 17.3) | 24.8  (24.4; 25.2) | 24.9  (24.4; 25.5) | 7.0  (6.7; 7.4) | 75.0  (74.5; 75.6) | 18.7  (18.5; 18.8) | 25.4  (25.1; 25.8) |
| Yes | 30.3  (27.9; 32.7) | 9.2  (8.0; 10.4) | 66.4  (64.0; 68.7) | 17.9  (17.4; 18.4) | 28.0  (26.4; 29.6) | 28.3  (26.2; 30.5) | 8.9  (7.6; 10.2) | 71.6  (69.5; 73.8) | 19.5  (18.8; 20.2) | 28.0  (26.4; 29.7) |
| **Parity**  **(number of previous children)** |  |  |  |  |  |  |  |  |  |  |
| 1 | 26.7  (26.0; 27.5) | 7.4  (7.0; 7.7) | 69.8  (69.0; 70.6) | 17.4  (17.2; 17.6) | 25.6  (25.1; 26.1) | 25.7  (25.0; 26.5) | 7.5  (7.0; 7.9) | 74.2  (73.5; 75.) | 19.0  (18.8; 19.3) | 26.2  (25.7; 26.7) |
| 2 | 25.1  (24.0; 26.1) | 6.7  (6.2; 7.1) | 71.5  (70.5; 72.5) | 16.9  (16.7; 17.2) | 24.4  (23.7; 25.1) | 24.9  (23.9; 25.9) | 7.1  (6.4; 7.8) | 75.1  (74.1; 76.0) | 18.3  (18.0; 18.6) | 25.1  (24.4; 25.7) |
| 3 or more | 24.7  (23.2; 26.1) | 6.6  (6.0; 7.3) | 71.9  (70.5; 73.2) | 17.1  (16.8; 17.4) | 24.6  (23;7; 25.5) | 24.2  (22.9; 25.5) | 6.5  (5.9; 7.1) | 75.8  (74.5; 77.0) | 18.5  (18.1; 18.9) | 25.0  (24.1; 25.9) |
| **Prematurity** |  |  |  |  |  |  |  |  |  |  |
| No | 26.0  (25.4; 26.6) | 7.1  (6.8; 7.4) | 70.6  (70.0; 71.1) | 17.3  (17.1; 17.4) | 25.2  (24.8; 25.6) | 25.4  (24.8; 26.0) | 7.3  (7.0; 7.7) | 74.6  (74.0; 75.1) | 18.8  (18.6; 19.0) | 25.8  (25.4; 26.2) |
| Yes | 25.0  (23.5; 26.4) | 6.4  (5.8; 6.9) | 71.6  (70.1; 73.0) | 16.8  (16.4; 17.1) | 24.0  (23.1; 24.8) | 23.9  (22.5; 25.2) | 6.2  (5.6; 6.7) | 76.0  (74.7; 77.4) | 18.3  (17.9; 18.7) | 24.5  (23.7; 25.4) |

FM (kg): fat mass in kilogram; FM (%): fat mass in percentage; LM (kg): lean mass in kilogram; LM (%): lean mass in percentage; TBM (kg): total body mass in kilogram. SD: Standard Deviation; 95%CI: Confidence interval

**Supplementary Table 2.** Mean of body composition measures according to independent variables in boys. 2015 Birth Cohort, Pelotas/RS, Brazil.

|  | **DEXA** | | | | | **BodPod** | | | | |
| --- | --- | --- | --- | --- | --- | --- | --- | --- | --- | --- |
|  | **FM** |  | **LM** |  | **TBM** | **FM** |  | **LM** |  | **TBM** |
|  | **%** | **Kg** | **%** | **Kg** | **Kg** | **%** | **Kg** | **%** | **Kg** | **Kg** |
|  | **β**  **(95%CI)** | **β**  **(95%CI)** | **β**  **(95%CI)** | **β**  **(95%CI)** | **β**  **(95%CI)** | **β**  **(95%CI)** | **β**  **(95%CI)** | **β**  **(95%CI)** | **β**  **(95%CI)** | **β**  **(95%CI)** |
| **Maternal age at delivery**  **(in years)** |  |  |  |  |  |  |  |  |  |  |
| < 20 | 20.9  (19.4; 22.4) | 5.8  (5.1; 6.5) | 75.6  (74.1; 77.1) | 18.6  (18.2; 19.0) | 25.3  (24.2; 26.3) | 17.8  (16.3; 19.2) | 5.0  (4.3; 5.6) | 82.2  (80.7; 83.7) | 20.5  (20.1; 20.9) | 25.5  (24.5; 26.5) |
| 20-34 | 21.5  (20.8; 22.2) | 6.1  (5.8; 6.5) | 75.0  (74.3; 75.7) | 18.7  (18.5; 18.9) | 25.7  (25.3; 26.2) | 19.2  (18.5; 19.9) | 5.7  (5.4; 6.1) | 80.8  (80.1; 81.5) | 20.6  (20.4; 20.9) | 26.3  (25.8; 26.7) |
| 35+ | 24.7  (23.1; 26.3) | 7.5  (6.7; 8.2) | 71.9  (70.3; 73.5) | 19.2  (18.8; 19.6) | 27.6  (26.5; 28.7) | 22.0  (20.4; 23.6) | 6.9  (6.1; 7.7) | 78.0  (76.3; 79.6) | 21.4  (20.9; 21.9) | 28.3  (27.1; 29.4) |
| **Maternal skin color** |  |  |  |  |  |  |  |  |  |  |
| White | 22.3  (21.6; 23.0) | 6.4  (6.1; 6.7) | 74.2  (73.6; 74.9) | 18.8  (18.6; 19.0) | 26.1  (25.6; 26.5) | 19.8  (19.1; 20.6) | 5.9  (5.5; 6.2) | 80.2  (79.4; 80.9) | 20.8  (20.6; 21.0) | 26.6  (26.1; 27.1) |
| Brown | 21.3  (19.6; 22.9) | 6.4  (5.5; 7.2) | 75.3  (73.6; 76.9) | 18.9  (18.5; 19.3) | 26.2  (25.0; 27.3) | 18.6  (16.9; 20.3) | 5.7  (4.9; 6.6) | 81.4  (79.7; 83.1) | 20.8  (20.3; 21.2) | 26.5  (25.3; 27.7) |
| Black | 20.5  (18.8; 22.2) | 5.8  (5.0; 6.5) | 76.0  (74.3; 77.6) | 18.7  (18.3; 19.1) | 25.3  (24.2; 26.4) | 18.5  (16.8; 20.2) | 5.3  (4.6; 6.1) | 81.5  (79.8; 83.2) | 20.6  (20.1; 21.0) | 25.9  (24.9; 27.0) |
| **Maternal education (years)** |  |  |  |  |  |  |  |  |  |  |
| 0-4 | 20.3  (18.0; 22.7) | 6.1  (4.9; 7.3) | 76.2  (73.9; 78.4) | 19.1  (18.6; 19.7) | 26.1  (24.4; 27.7) | 17.8  (15.4; 20.1) | 5.5  (4.3; 6.7) | 82.2  (79.9; 84.6) | 21.0  (20.3; 21.7) | 26.5  (24.8; 28.3) |
| 5-8 | 20.3  (19.2; 21.5) | 5.7  (5.1; 6.2) | 76.1  (75.0; 77.3) | 18.6  (18.3; 19.0) | 25.1  (24.3; 25.9) | 17.9  (16.7; 19.1) | 5.3  (4.7; 5.9) | 82.0  (80.8; 83.2) | 20.6  (20.2; 20.9) | 25.7  (24.9; 26.5) |
| 9-11 | 22.4  (21.4; 26.4) | 6.5  (6.0; 7.0) | 74.1  (73.2; 75.1) | 18.9  (18.6; 19.1) | 26.2  (25.6; 26.9) | 19.8  (18.8; 20.9) | 5.9  (5.4; 6.4) | 80.1  (76.3; 77.7) | 20.7  20.4; 21.0) | 26.6  (25.9; 27.3) |
| 12+ | 22.8  (21.8; 23.9) | 6.6  (6.1; 7.1) | 73.7  (72.7; 74.8) | 18.7  (18.4; 19.0) | 26.2  (25.5; 27.0) | 20.6  (19.6; 21.7) | 6.1  (5.6; 6.7) | 79.5  (78.4; 80.5) | 20.8  (20.5; 21.1) | 26.9  (26.2; 27.6) |
| **Family income (in quintiles)** |  |  |  |  |  |  |  |  |  |  |
| 1^st^ (poorest) | 20.7  (19.3; 22.1) | 5.9  (5.2; 6.6) | 75.8  (74.4; 77.1) | 18.7  (18.3; 19.0) | 25.4  (24.5; 26.4) | 18.6  (17.2; 20.0) | 5.4  (4.8; 6.1) | 81.4  (80.0; 82.8) | 20.6  (20.2; 20.9) | 26.0  (25.1; 27.0) |
| 2^nd^ | 20.5  (19.2; 21.8) | 5.7  (5.1; 6.3) | 76.0  (74.7; 77.3) | 18.6  (18.3; 19.0) | 25.1  (24.3; 26.0) | 18.5  (17.2; 19.7) | 5.3  (4.7; 5.8) | 81.5  (80.2; 82.8) | 20.5  (20.1; 20.8) | 25.7  (24.9; 26.6) |
| 3^rd^ | 23.6  (22.2; 25.0) | 7.0  (6.4; 7.7) | 73.0  (71.7; 74.3) | 18.9  (18.5; 19.3) | 26.9  (25.8; 27.9) | 21.1  (19.6; 22.5) | 6.8  (6.0; 7.6) | 78.9  (77.5; 80.3) | 21.1  (20.6; 21.5) | 27.6  (26.5; 28.6) |
| 4^th^ | 22.7  (21.4; 24.1) | 6.6  (6.0; 7.3) | 73.8  (72.5; 75.1) | 18.9  (18.6; 19.2) | 26.4  (25.5; 27.3) | 19.9  (18.6; 21.3) | 5.9  (5.2; 6.5) | 80.1  (78.8; 81.5) | 20.8  (20.4; 21.2) | 26.7  (25.8; 27.6) |
| 5^th^ (richest) | 21.8  (20.6; 23.1) | 6.2  (5.6; 6.7) | 74.7  (73.4; 76.0) | 18.8  (18.5; 19.1) | 25.8  (25.0; 26.7) | 19.2  (17.9; 20.5) | 5.6  (5.1; 6.2) | 80.7  (79.5; 82.0) | 20.8  (20.4; 21.2) | 26.3  (25.5; 27.1) |
| **Pre-pregnancy BMI (kg/m^2^)** |  |  |  |  |  |  |  |  |  |  |
| < 25.0 | 19.3  (18.5; 20.0) | 5.0  (4.7; 5.3) | 77.2  (76.5; 77.9) | 18.3  (18.1; 18.5) | 24.2  (23.7; 24.6) | 17.1  (16.3; 17.9) | 4.8  (4.4; 5.1) | 82.9  (82.1; 83.6) | 20.1  (19.9; 20.3) | 24.7  (24.3; 25.2) |
| 25.0 until < 30.0 | 22.3  (21.2; 23.4) | 6.4  (5.9; 6.9) | 74.2  (73.2; 75.3) | 19.0  (18.7; 19.3) | 26.3  (25.6; 27.0) | 19.8  (18.7; 21.0) | 5.8  (5.3; 6.3) | 80.2  (79.1; 81.3) | 20.9  (20.6; 21.2) | 26.7  (26.0; 27.4) |
| ≥ 30.0 | 27.2  (25.8; 28.6) | 8.8  (8.1; 9.6) | 69.5  (68.1; 70.9) | 19.5  (19.1; 19.9) | 29.3  (28.1; 30.4) | 24.5  (23.1; 25.9) | 8.1  (7.4; 8.9) | 75.5  (74.0; 76.9) | 21.8  (21.3; 22.3) | 30.0  (28.8; 31.1) |
| **Gestational arterial**  **hypertension** |  |  |  |  |  |  |  |  |  |  |
| No | 21.1  (20.4; 21.8) | 5.9  (5.6; 6.2) | 75.4  (74.7; 76.1) | 18.7  (18.5; 18.9) | 25.5  (25.1; 26.0) | 18.7  (18.0; 19.4) | 5.5  (5.2; 5.8) | 81.3  (80.6; 81.9) | 20.6  (20.4; 20.8) | 26.0  (25.5; 26.4) |
| Yes | 24.0  (22.8; 25.3) | 7.3  (6.6; 7.9) | 72.6  (71.4; 73.8) | 19.0  (18.7; 19.3) | 27.2  (26.3; 28.1) | 21.5  (20.2; 22.8) | 6.7  (6.1; 7.3) | 78.6  (77.3; 79.8) | 21.2  (20.8; 21.6) | 27.9  (27.0; 28.8) |
| **Gestational diabetes** |  |  |  |  |  |  |  |  |  |  |
| No | 21.4  (20.7; 22.0) | 6.0  (5.8; 6.3) | 75.1  (74.5; 75.7) | 18.7  (18.6; 18.9) | 25.6  (25.2; 26.0) | 18.9  (18.3; 19.6) | 5.6  (5.3; 5.9) | 81.1  (80.4; 81.7) | 20.6  (20.5; 20.8) | 26.1  (25.7; 26.5) |
| Yes | 26.2  (24.1; 28.4) | 8.4  (7.3; 9.6) | 70.4  (68.4; 72.5) | 19.3  (18.7; 19.9) | 28.6  (27.0; 30.3) | 23.7  (21.7; 25.8) | 7.8  (6.7; 8.9) | 76.2  (74.2; 78.3) | 21.6  (21.0; 22.1) | 29.4  (27.8; 31.0) |
| **Parity**  **(number of previous children)** |  |  |  |  |  |  |  |  |  |  |
| 1 | 22.2  (21.4; 23.1) | 6.4  (6.0; 6.8) | 74.3  (73.5; 75.1) | 18.8  (18.5; 19.0) | 26.0  (25.5; 26.6) | 19.6  (18.7; 20.4) | 5.7  (5.4; 6.1) | 80.4  (79.5; 81.2) | 20.7  (20.4; 20.9) | 26.4  (25.8; 26.9) |
| 2 | 22.1  (21.0; 23.2) | 6.4  (5.9; 6.9) | 74.4  (73.4; 75.5) | 18.7  (18.4; 19.0) | 26.0  (25.3; 26.8) | 20.2  (19.1; 21.3) | 6.2  (5.7; 6.8) | 79.8  (78.7; 80.9) | 20.8  (20.5; 21.1) | 26.9  (26.1; 27.6) |
| 3 or more | 20.6  (19.2; 22.0) | 5.8  (5.2; 6.5) | 75.8  (74.5; 77.2) | 18.9  (18.6; 19.2) | 25.6  (24.7; 26.5) | 17.9  (16.5; 19.3) | 5.3  (4.6; 6.0) | 82.1  (80.7; 83.5) | 20.8  (20.4; 21.2) | 26.1  (25.1; 27.1) |
| **Prematurity** |  |  |  |  |  |  |  |  |  |  |
| No | 22.1  (21.5; 22.8) | 6.4  (6.1; 6.7) | 74.4  (73.8; 75.1) | 18.8  (18.7; 19.0) | 26.1  (25.7; 26.6) | 19.8  (19.1; 20.5) | 5.9  (5.6; 6.3) | 80.2  (79.5; 80.9) | 20.8  (20.6; 21.0) | 26.6  (26.2; 27.1) |
| Yes | 20.4  (18.9; 21.9) | 5.5  (4.9; 6.2) | 76.1  (74.6; 77.5) | 18.5  (18.2; 18.9) | 24.9  (24.1; 25.8) | 17.5  (16.1; 19.0) | 5.0  (4.4; 5.6) | 82. 4  (81.0; 83.9) | 20.6  (20.1; 21.0) | 25.4  (24.6; 26.3) |

FM (kg): fat mass in kilogram; FM (%): fat mass in percentage; LM (kg): lean mass in kilogram; LM (%): lean mass in percentage; TBM (kg): total body mass in kilogram. SD: Standard Deviation; 95%CI: Confidence interval

**Supplementary Table 3.** Mean of anthropometric indicators according to body composition exams. 2015 Birth Cohort, Pelotas/RS, Brazil.

| **Anthropometric indicators** | **BodPod** | | | | **DXA** | | | |
| --- | --- | --- | --- | --- | --- | --- | --- | --- |
|  | **No** | | **Yes** | | **No** | | **Yes** | |
|  | **Mean (SD)** | **95%CI** | **Mean (SD)** | **95%CI** | **Mean (SD)** | **95%CI** | **Mean (SD)** | **95%CI** |
| Weight-for-age z-score | 0.98 (1.6) | 0.89; 1.06 | 0.96 (1.5) | 0.90; 1.03 | 0.97 (1.6) | 0.89; 1.06 | 0.97 (1.5) | 0.90; 1.03 |
| Height-for-age z-score | 0.49 (1.1) | 0.43; 0.55 | 0.48 (1.1) | 0.43; 0.52 | 0.48 (1.2) | 0.42; 0.54 | 0.49 (1.1) | 0.44; 0.53 |
| BMI-for-age z-score | 0.92 (1.7) | 0.84; 1.01 | 0.93 (1.6) | 0.86; 1.00 | 0.93 (1.6) | 0.84; 1.02 | 0.93 (1.6) | 0.86; 0.99 |

95%CI: Confidence interval; SD: Standard Deviation
